# Miniature Fourier Transform Spectrometer Based on Thin-Film Lithium Niobate

**DOI:** 10.3390/mi14020458

**Published:** 2023-02-15

**Authors:** Lichao Zhang, Guangyang Gou, Jiamin Chen, Wangzhe Li, Weichao Ma, Ruoming Li, Junming An, Yue Wang, Yuanyuan Liu, Wei Yan, Tianjun Ma, Chunxiu Liu, Jianjun Cheng, Zhimei Qi, Ning Xue

**Affiliations:** 1 State Key Laboratory of Transducer Technology, Aerospace Information Research Institute, Chinese Academy of Sciences, Beijing 100190, China; 2 School of Electronic, Electrical and Communication Engineering, University of Chinese Academy of Sciences, Beijing 100049, China; 3 Institute of Semiconductors, Chinese Academy of Sciences, Beijing 100083, China; 4 Center of Materials Science and Optoelectronics Engineering, University of Chinese Academy of Sciences, Beijing 100049, China; 5 School of Integrated Circuit, Quanzhou University of Information Engineering, Quanzhou 362000, China

**Keywords:** lithium niobate, Fourier transform spectrometer, electro-optical modulation

## Abstract

A miniature Fourier transform spectrometer is proposed using a thin-film lithium niobate electro-optical modulator instead of the conventional modulator made by titanium diffusion in lithium niobate. The modulator was fabricated by a contact lithography process, and its voltage-length and optical waveguide loss were 2.26 V·cm and 1.01 dB/cm, respectively. Based on the wavelength dispersion of the half-wave voltage of the fabricated modulator, the emission spectrum of the input signal was retrieved by Fourier transform processing of the interferogram, and the analysis of the experimental data of monochromatic light shows that the proposed miniaturized FTS can effectively identify the input signal wavelength.

## 1. Introduction

Due to the ability to detect the characteristics of optical signals, spectrometers are widely used in many fields, such as space exploration [1], nanotechnology [2], bioengineering [3], modern medicine [4], meteorological monitoring [5], resource exploration [6], environmental monitoring [7] and life sciences [8]. With the development of science and technology, there is an increasingly urgent need for spectrometers to move from the traditional benchtop to miniaturization.

In recent years, the development of integrated optics has promoted the development of a miniature Fourier transform spectrometer (FTS) based on the electro-optical effect in lithium niobate (LN) to achieve a tunable optical pathlength difference (*OPD*) has received great attention.

In 1893, German physicist Pockels discovered the linear electro-optical effect, which revealed that the refractive index of certain crystals varies linearly with an applied voltage. As a material with a linear electro-optic effect, LN is a good material platform for miniature FTS because of its relatively large electro-optic coefficient, wide optical transparency window (0.4~5 μm), and low power consumption. In 2002, Howard constructed the first FTS based on bulk LN crystal [9]. Then a miniature FTS was introduced by Bentini et al. in 2007, which was achieved by utilizing high-energy ion implantation in LN [10]. In 2014, Li et al. proposed a miniature FTS, which is based on a conventional electro-optical modulator made by diffusing titanium into LN [11]. However, the electro-optical modulation efficiency of titanium diffused LN waveguide is low. Around 2016, thin film lithium niobate (TFLN) wafers were commercially produced, giving rise to a series of studies based on TFLN [12,13,14,15,16]. TFLN waveguides are mostly formed by dry etching, allowing for high electro-optical modulation efficiency. There are many explorations of an electro-optical modulator based on TFLN, but there are few studies combining it with FTS. In 2019, Grange et al. combined the principles of LN electro-optical modulation and standing wave interference to propose a hybrid integrated FTS based on a TFLN-silicon nitride platform [17].

In this work, we present a miniature FTS based on a TFLN electro-optical modulator which was fabricated by a contact lithography process. Due to the half-wave voltage V_π_ dependence on wavelength, the emission spectrum of the input signal is retrieved by the Fourier transform of the measured temporal interferogram. This work is of great value for the miniaturization of FTS.

This paper is organized as follows: Section 2 describes the detailed design of FTS and simulation of the modulator; then the fabrication, measurement, and analysis of results are presented in Section 3.

## 2. Design and Simulation

In this section, the general description of the device is given and then simulations are performed based on the relevant parameters to determine the dimensions of the modulation area.

**Figure 1:**
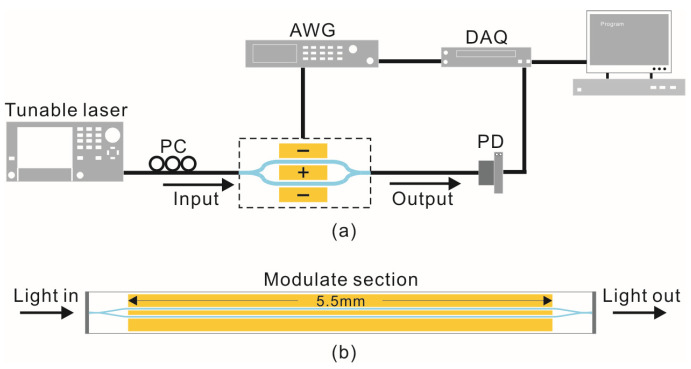
(**a**) The schematic of the FTS; (**b**) The schematic of the TFLN electro-optical modulator.

The schematic diagram of the miniature FTS based on lithium niobate on insulator (LNOI) described in this paper is shown in Figure 1a, which consists of an electro-optical modulator, an arbitrary waveform generator (AWG), a photodetector (PD), a polarization controller (PC), a data acquisition card (DAQ), a signal processing unit, and input and output optical fibers. The core component is an electro-optical modulator based on LNOI, which consists of a Mach-Zehnder interferometer (MZI) and traveling wave electrodes (TWE), as shown in Figure 1b. Firstly, the optical signal is coupled into the MZI through the input fiber and propagates in the two arms of the MZI. At the same time, a modulating voltage is generated by the voltage generator and is applied to the TWEs on both sides of the waveguide, which leads to an opposite change in the refractive index of the two LN arms, thus modulating the *OPD* between both arms. Subsequently, the two optical signals interfere at the Y-branch combiner, and the interferometric optical signal is captured by the PD through the output fiber, and then is converted into an electrical signal at the output to the DAQ. Simultaneously, the modulating voltage is also sampled by the DAQ. Based on the Fourier transform principle and the relevant algorithms [18], the Fourier transform is performed on the acquired signals through the signal processing unit, and the emission spectrum of the input signal can be obtained. Among them, the V_π_ of the electro-optical modulator has a great influence on the performances of the miniature FTS.

The configuration and dimensions of the electro-optical modulator are shown in Figure 1b. The chip size is 7.5 mm × 0.25 mm, the modulating arm length of the interferometer is 5.5 mm, and the width of both waveguides is 1.1 µm. Considering that the input and output fiber waist diameters are 4 μm, the input port of the waveguide is designed with a tapered shape with the width gradually decreasing from 6 μm to 1.1 μm in order to make the input optical signal better coupled into the waveguide through the fiber, and the length of the taper is 500 μm. The output port of the waveguide is similarly designed. The two modulating arms are flanked by TWEs.

**Figure 2:**
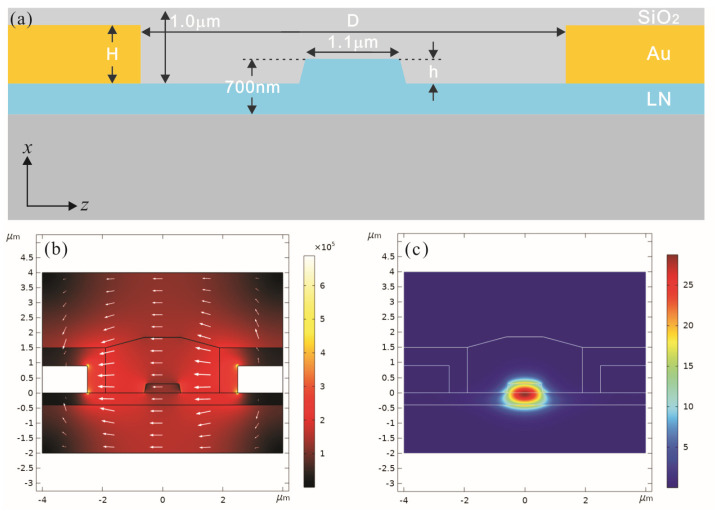
(**a**) The cross−sectional structure and dimensions of the modulation area; (**b**) The electrostatic field of two electrodes; (**c**) The optical field of the transverse electric (TE) mode.

Figure 2a shows the cross-sectional structure of the modulated region. From top to bottom, are the SiO_2_ cladding layer, the two gold electrodes, the x-cut LNOI ridge waveguide, the SiO_2_ buried layer, and the silicon substrate. The optical signals are confined in the ridge waveguide for transmission, and the sidewall roughness of the ridge waveguide is greatly affected by fabrication conditions. Considering that contact lithography is used in this research, in the simulations, the upper width of the ridge waveguide is set to 1.1 μm. When the modulating voltage is applied to the gold electrodes on both sides of the ridge waveguide, it will result in a change in the refractive index of the ridge waveguide. The electric field applied to the two arms of the interferometer in opposite directions causes opposite changes in the refractive index of the two arms, resulting in the *OPD* at the end of the interferometer. For the electro-optical modulator, the V_π_ and optical waveguide loss are greatly influenced by the gap D of the two gold electrodes, the etching depth h of the ridge waveguide, and the height of gold electrodes H, as shown in Figure 2a. Related studies have shown [19] that the larger the gap D and the larger the etching depth h, the larger the V_π_ and the smaller the optical loss caused by metal absorption. In order to obtain a relatively low V_π_ and relatively small optical waveguide loss, with reference to the results of the literature [19] and a series of related simulations using COMSOL Multiphysics, in our research, the gap D is set as 5 µm, the etching depth h equals 300 nm and the height of gold electrodes H equals 900 nm.

Based on the determined dimensions, the simulations were carried out by COMSOL Multiphysics, and the results are shown in Figure 2b,c, including the optical field of the simulated transverse electric (TE) mode and the electrostatic field of the gold electrodes, with a calculated electro-optic overlap integral factor of 0.5401. Then the V_π_ and optical waveguide loss of the electro-optical modulator can be calculated based on COMSOL simulations. At λ = 1550 nm, the corresponding V_π_ is 4.08 V, and the optical waveguide loss is 0.38 dB/cm.

## 3. Fabrication of the Device and Analysis of Measurement Results

### 3.1. Fabrication and Measurement of the Device

**Figure 3:**
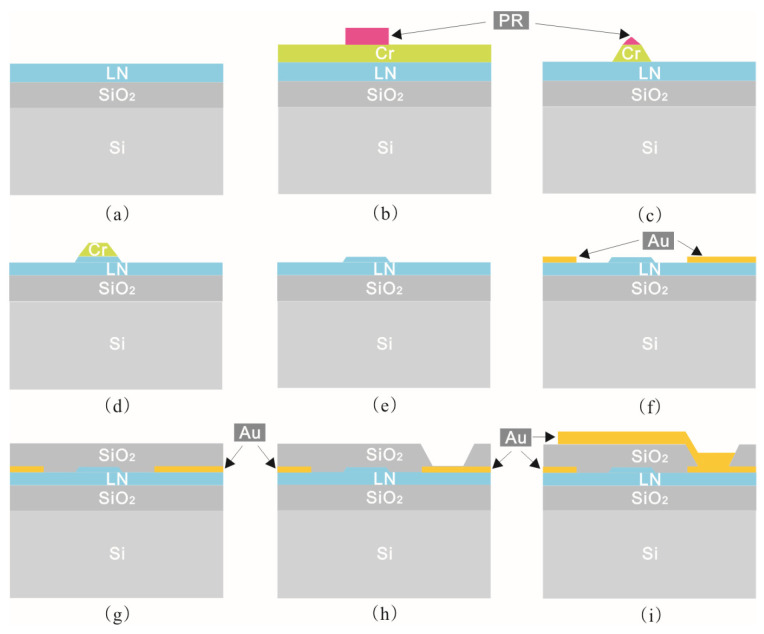
Schematic diagram of the fabrication process of the TFLN electro-optical modulator. (**a**) An x-cut LNOI substrate is (**b**) coated with 80 nm thick Cr by magnetron sputter. Then a 1.2-µm-thick negative PR is used to define the waveguide pattern. (**c**) The patterns are then transferred into the Cr layer using ICP etcher, and (**d**) subsequently into the LN thin film using Ar and SF6. (**e**) The residue mask materials are removed in cerium ammonium nitrate. (**f**) Bottom metal electrodes (15 nm-thick Cr/900 nm-thick Au) are formed using a standard lift-off process. (**g**) A 1 μm thick silica cladding layer is then deposited by PECVD. (**h**) Via windows are opened by wet etching. (**i**) A second lift-off process is performed to produce the top electrodes.

We use contact lithography to prepare the electro-optic modulator chip, and the fabrication process is shown in Figure 3. First, an 80 nm thick Chromium layer (Cr) is sputtered on the top surface of the exposed LNOI by magnetron sputtering, which is used as a hard mask for the etching process. A 1.2-μm-thick negative photoresist (PR) is then used to define the waveguide pattern. Next, the patterns are transferred into a Cr layer using an inductively coupled plasma (ICP) etcher, and subsequently into the TFLN by an optimized dry ICP etching process based on Ar and SF_6_. The physical and chemical reactions are carried out simultaneously during the TFLN etching process, and the etching speed is 0.32 nm/s. Then the residual Cr mask is removed using cerium ammonium nitrate. The metal electrode (15 nm-thick Cr/900 nm-thick Au) is then fabricated and formed by a standard lift-off process. A 1 μm thick silica cladding is then deposited by plasma enhanced chemical vapor deposition (PECVD). Then windows are made in the silica cladding to facilitate the connection of the bottom electrode to the top electrode afterward, and finally, a second lift-off process is performed to produce the top electrode.

**Figure 4:**
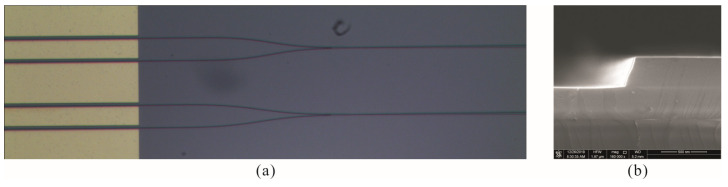
(**a**) Microscopic image of the part of the modulator; (**b**) The SEM image of the etched waveguide cross section.

Figure 4 shows the microscope image of a part of the fabricated modulator and the scanning electron microscope (SEM) image of a cross section of the waveguide. The upper width of the ridge waveguide is measured to be about 1.1 μm, the sloping sidewall of the waveguide is about 75° from horizontal, and the bottom width of the ridge waveguide is about 1.2 μm.

**Figure 5:**
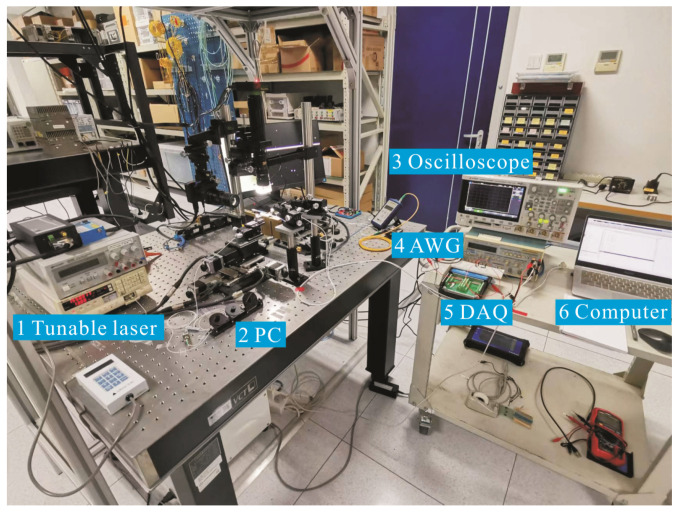
The measurement setup.

The measurement setup is shown in Figure 5. The input optical signal is generated by a tunable laser and coupled into the electro-optical modulator through the input fiber and PC. After modulation by the modulating voltage, the optical signals in the two arms interfere at the Y-branch combiner, and the interfering optical signals are captured by the PD through the output fiber and then are converted into electrical signals, and the DAQ is set at the end to capture the electrical signals. Table 1 compares the fabricated TFLN modulator in this work with the one obtained in the [19]. They have similar structures and working principles.

### 3.2. Analysis of Measurement Results

In this section, the principle of optical signal identification based on FTS is described, and then the identification of the input optical signal based on the experimental data is conducted.

The change of *OPD* in the proposed miniature FTS is achieved by the electro-optical effect in LN. The opposite refractive index change caused by the modulating voltage V(t) will produce *OPD* although the physical paths of the two arms are the same. The *OPD* between the two arms can be expressed as

(1) $$OPD=\frac{n_{e}^{3}\left(\lambda \right)\gamma_{33}\left(\lambda \right)\Gamma \left(\lambda \right)L}{D}V\left(t\right)$$

where *D* is the gap between the two gold electrodes, *L* is the arm length, *n_e_*(*λ*), *γ*_33_(*λ*), Γ(*λ*) are the parameters related to modulator configuration and optical signal wavelength *λ*. The half-wave voltage *V_π_*(*λ*) of the electro-optical modulator can be expressed as

(2) $$V_{\pi }\left(\lambda \right)=\frac{\lambda D}{2n_{e}^{3}\left(\lambda \right)\gamma_{33}\left(\lambda \right)\Gamma \left(\lambda \right)L}$$

Substituting Equation (2) into Equation (1), we can get

(3) $$OPD=\frac{\lambda V\left(t\right)}{2V_{\pi }\left(\lambda \right)}$$

Defining *g*(*λ*) = 1/(2*V_π_* (*λ*)), Equation (3) can be rewritten as

(4) OPD=λV(t)g(λ)

For a fabricated electro-optical modulator, *L* and *D* are all constant, and the parameters n_e_(*λ*), *γ*_33_(*λ*) and Γ(*λ*) have definite values when the input optical signal wavelength *λ* is determined. Then according to Equation (2), *V_π_*(*λ*), and *g*(*λ*) can be determined, and *OPD* is only related to the applied modulating voltage *V*(*t*) according to Equation (1).

The modulating voltage will cause the change of *OPD*, thus causing the change of the interferometric light intensity *I*. *I* varies with the modulating voltage *V* and can be expressed as follows, according to the FTS theory [11],

(5) $$I\left(V\right)=\overset{\infty }{\underset{0}{\int }}A\left(\lambda \right)\cos\left[2\pi \frac{OPD}{\lambda }\right]d\lambda$$

where *A*(*λ*) is the light intensity in the emission spectrum of the input signal. Equations (4) and (5) can be rewritten as

(6) $$I\left(V\right)=\overset{\infty }{\underset{0}{\int }}A\left(\lambda \right)\cos\left[2\pi V\left(t\right)g\left(\lambda \right)\right]d\lambda$$

To facilitate the Fourier transform, Equation (6) is rewritten as [11]

(7) $$I\left(V\right)=\overset{\infty }{\underset{0}{\int }}A^{\prime }\left(g\right)\cos\left[2\pi V\left(t\right)g\right]dg$$

Fourier transform of Equation (7) gives

(8) $$A\left(g\right)=\overset{\infty }{\underset{-\infty }{\int }}I\left(V\right)\exp\left(-j2\pi gV\left(t\right)\right)dV$$

It is worth noting that due to the presence of a breakdown electric field (~10 V/μm) in LN, the range of modulating voltage is limited, then Equation (8) can be rewritten as

(9) $$A_{0}\left(g\right)=\overset{V_{max}}{\underset{-V_{max}}{\int }}I\left(V\right)\exp\left(-j2\pi gV\right)dV$$

where *V_max_* is the maximum modulating voltage, and *A*_0_(*g*) is the spectral intensity distribution with g. Since the collected data of the modulating voltage *V*(*t*) and the interferometric light intensity *I* are discrete sequences, the discrete Fourier transform is used, which is expressed as [20]

(10) $$A_{0}\left(g\right)=\overset{N_{s}-1}{\underset{i=0}{\sum }}I_{i}\exp\left(-j2\pi gV_{i}\right)dV_{i},\;\;dV_{i}=\left(V_{i+1}-V_{i-1}\right)/2$$

*A*_0_(*g*) can be obtained through the discrete Fourier transform. Obtaining *A*(*λ*) from *A*_0_(*g*) also requires determining the dispersion relation between the half-wave voltage *V_π_*(*λ*) and the wavelength *λ*.

For the fabricated FTS, the dependence of *V_π_* on wavelength *λ* can be determined by experimentally measuring a series of input optical signals of different *λ*, and the study shows that the function has monotonicity [20]. By using the measured dispersion of *V_π_*(*λ*) and the formula *g* = [2*V_π_*(*λ*)] − 1, the plot of *A*_0_(*g*) against *g* can be converted into the wanted laser power spectrum *A*(*λ*). A more detailed discussion of the above theory can be found in the literature [11]. Based on the above principles, specific signal processing algorithms are written to analyze the experimental data of the fabricated miniature FTS, then the identification of the input optical signal can be conducted.

**Figure 6:**
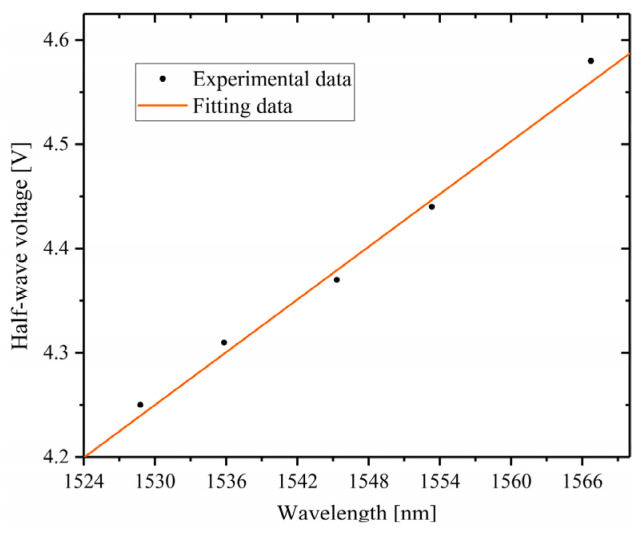
The half-wave voltage dispersion.

In order to obtain the half-wave voltage *V_π_*(*λ*) as a function of wavelength *λ*, a series of optical signals with different wavelengths (1528.8 nm, 1535.8 nm, 1545.3 nm, 1553.3 nm, 1566.7 nm) are fed in the TFLN electro-optic modulator, and the *V_π_* corresponding to a certain wavelength can be obtained by the intensity-versus-voltage interferogram, then a series of the values of *V_π_* and *λ* can be obtained, as shown by the black dots in Figure 6. The half-wave voltage dispersion can be obtained by fitting the measured multiple wavelengths to the half-wave voltage data, as shown in Figure 6. The fitting function is expressed as

*V_π_*(*λ*) = 0.00853 × *λ* − 8.80257 (11)

**Figure 7:**
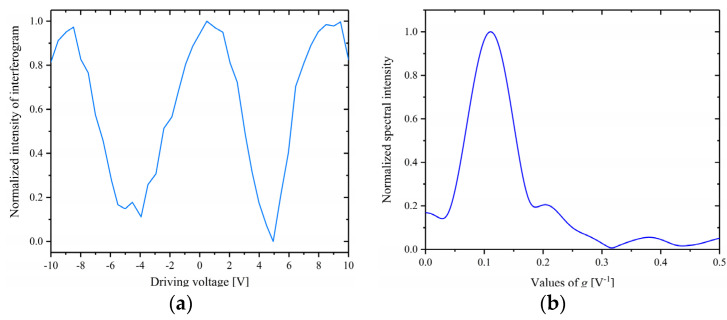
(**a**) The normalized intensity−versus−voltage interferogram; (**b**) The normalized spectral intensity distributed with g.

In order to verify the spectral detection capability of the miniature FTS proposed in this paper, the light signal with a wavelength of 1561.828 nm was fed into the miniature FTS as the light source to be measured, and the intensity-versus-voltage interferogram was obtained, as shown in Figure 7a. It can be read from Figure 7a that the half-wave voltage is 4.51 V, corresponding to a voltage-length product of 2.26 V·cm. After Fourier transforming of the interferogram, the plot of normalized *A*_0_(*g*) against g was obtained (as shown in Figure 7b), on which the plot of *A*(*λ*) against λ can be obtained utilizing the formula *g* = [2*V_π_*(*λ*)] − 1 and the Equation (11). It can be deduced that the main peak is located at 1561.46 nm, which is very close to the wavelength of the signal to be measured (1561.828 nm), indicating that the proposed miniature FTS can accurately identify the wavelength of the input signal.

## 4. Conclusions

A miniature FTS is proposed using a shallowly etched TFLN electro-optical modulator instead of the conventional modulator made by titanium diffusion in LN. The modulator was fabricated by a contact lithography process and ICP dry etching process, which can reduce the cost of the device. Its voltage-length product and optical waveguide loss were 2.26 V·cm and 1.01 dB/cm, respectively, and the measured results agree well with simulated results, which indicates that the simulation is a good guide to improve the modulator performance. The half-wave voltage as a function of wavelength was experimentally determined. Based on the wavelength dispersion of half-wave voltage, the emission spectrum of the input signal was retrieved by Fourier transform processing of the interferogram. A signal with a wavelength of 1561.828 nm was input to the proposed miniaturized FTS, and the signal spectrum with a peak located at 1561.46 nm was obtained with the help of the half-wave voltage dispersion equation and Fourier transform. This shows that the proposed miniaturized FTS can effectively identify the input signal wavelength. We believe that the miniaturized FTS presented in this paper can be a promising candidate for compact FTS.

## Figures and Tables

**Table 1:** Performance comparison of TFLN modulators.

Voltage-Length Product	Pattern Definition	Half-Wave Voltage	Gap	Etching Depth	Propagation Loss
1.75 V·cm	Photolithography	3.5 V	5 µm	0.3 µm	0.7 dB/cm
2.26 V·cm	Photolithography	4.51 V	5 µm	0.3 µm	1.01 dB/cm

## Data Availability

Data are available upon reasonable request from the corresponding author.

## Abbreviations

FTS	Fourier transform spectrometer
LN	lithium niobate
*OPD*	optical pathlength difference
TFLN	thin film lithium niobate
LNOI	lithium niobate on insulator
AWG	arbitrary waveform generator
PD	photodetector
PC	polarization controller
DAQ	data acquisition card
MZI	Mach-Zehnder interferometer
TWE	traveling wave electrodes
TE	transverse electric
PR	photoresist
ICP	inductively coupled plasma
PECVD	plasma enhanced chemical vapor deposition
SEM	scanning electron microscope
